# Supplementary material for: Is Cell-Free DNA Testing in Pancreatic Ductal Adenocarcinoma Ready for Prime Time?
Source: Cancers (Basel). 2022 Jul 15;14(14):3453. doi: 10.3390/cancers14143453 (PMC9322623; doi:10.3390/cancers14143453)
Supplement: Supplementary file 1 [file cancers-14-03453-s001.zip › cancers-1796927-Supplementary.pdf]

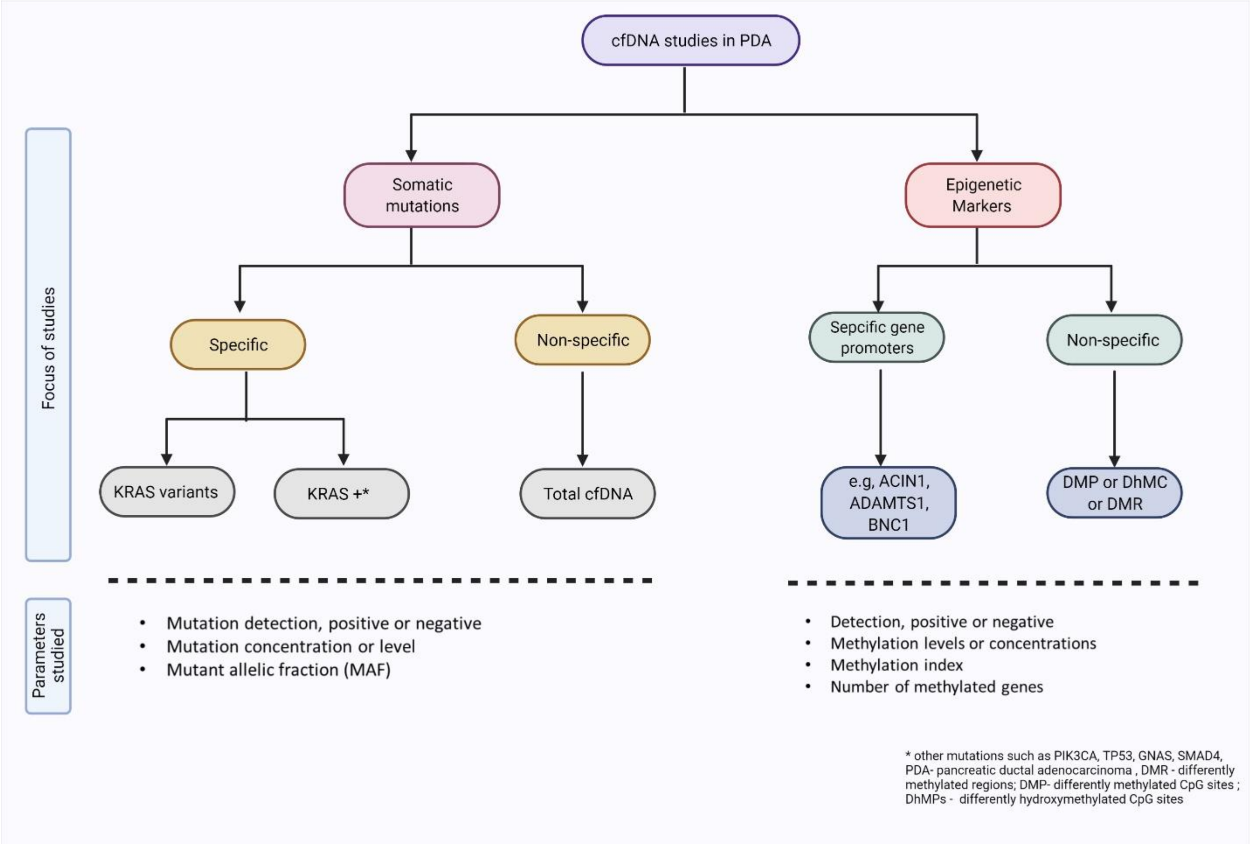

Supplementary Figure S1. Summary of cfDNA studies in PDA

Overview of parameters examined in blood cfDNA studies as related to somatic mutations and epigenetic markers in patients with PDA.

Supplementary Table S1. Summary of studies examining somatic mutations for diagnosis of PDA with concordance rate compared to tissue.

Study	Method of Detection	Stage of Disease	Target	Concordance Rate vs Tissue
Berger, *et al.* [134]	ddPCR	Serous Cystadenoma, IPMN, metastatic PDA	Total Concentration, GNAS, KRAS	Not available
Brychta, *et al.* [35]	ddPCR	Early-Stage Disease (Stage I or II)	KRAS	90% of KRAS mutations could be detected in cfDNA
Le Calvez-Kelm, *et al.* [135]	NGS	All stages of PDA and chronic pancreatitis	KRAS	Not available
Cohen, *et al.* [136]	PCR	All stages of PDA	KRAS	30% of KRAS mutations could be detected in cfDNA
Cohen, *et al.* [36]	CancerSEEK (PCR combined with protein identification)	Resectable PDA	16 gene panel	100% of KRAS mutations could be detected in cfDNA
Berger, *et al.* [137]	Fluorometric analysis of cfDNA	All stages PDA, IPMN, chronic pancreatitis	Total concentration, THBS2	Not available
Liu, *et al.* [138]	NGS	All stages PDA, IPMN	791gene panel set including KRAS, EGFR and TP53	>94% of KRAS mutations could be detected in cfDNA

Polymerase Chain Reaction (PCR), PCR-Restriction Fragment Length Polymorphism (RLFP), Digital Droplet PCR (ddPCR), beads, emulsion, amplification, and magnetics PCR (BEAM-PCR), Next generation sequencing (NGS); Intraductal Papillary Mucinous Neoplasm (IPMN); pancreatic ductal adenocarcinoma (PDA)

Supplementary Table S2: Risk stratification based on 28-gene panel and performance status in PDA

	**Univariate screening HR (95% CI)**	**Using prediction model HR (95% CI)**
All stages	BNC1 - 2.10 (1.36-3.25)<br>GSTP1 - 6.91 (2.08-22.96)<br>MLH1 - 1.85 (1.03-3.32)<br>SFRP1- 2.11 (1.38-3.23)<br>SEPT9v2 - 2.37 (1.32-4.27)<br>SST- 1.63 (1.06-2.51)<br>TFP12- 2.22 (1.34-3.68)<br>WNT5A- 2.32 (1.09-4.94)	BNC1 2.00 (1.26-3.18)<br>GSTP1 - 9.55 (2.70-33.82)<br>TFP12 - 2.52 (1.42-4.47)<br>SFRP1 - 1.94 (1.24-3.02<br>SFRP2- 0.45 (0.27-0.73)<br>ASA 3- 3.34 (1.91-5.84)<br>PS > 0 vs PS = 0 2.49 (1.61-3.84)
Stage I/II	SFRP2 0.31 (0.14-0.71)<br>CDK2NA - 9.24 (95% CI; 1.03-82.68)	SFRP2- 0.18 (0.07-0.45)<br>MESTv2 - 2.39 (0.97-5.94)<br>ASA 3= 14.13 (4.56-43.81)
Stage III	WNT5A (III) - 7.05 (0.97-51.19)	Not done
Stage IV	BMP3 - 3.21 (1.58-6.53)<br>SFRP1 - 4.57(2.02-10.34)<br>TFP12 - 2.590.01(1.25-5.39)	BMP3 - 2.65 (1.11-6.29)<br>MGMT- 2.11 (0.57-7.87)<br>NPTX2 - 0.45 (0.17-1.18)<br>SFRP1 2.77 (1.15-6.67)

HR-Hazards Ratio; OR-Odds Ratio; CI – confidence interval

Supplementary Table S3: Outcomes in prominent adjuvant therapy trials in pancreatic ductal adenocarcinoma

TRIAL	EXPERIMENTAL (months)	COMPARITIVE (months)	MEDIAN F/U (months)	Notes
ESPAC 4<br>Gem/cap vs Gem	30.2	29.7	43.2	5-yr survival rate, 28 % vs 17%
PRODIGE-4/ACCORD 11<br>FOFIRINOX vs Gem	54.4	35	33.6	3-yr survival rate, 63.4 % vs 48.6%
Gem vs Obs	22.1	20.2	53	Recurrence rate – 74% vs 92%<br>No Sig diff in OS
ESPAC 3<br>5FU vs Gem	23	23.6	34.2	No Sig diff in PFS
CONKO-005<br>Gem/Erl vs Gem	24.6	26.5	53	No Sig diff in PFS and OS

Gem- gemcitabine; Cap – capecitabine, Obs – best supportive care, 5FU – 5-flurouracil, Erl- erlotinib, Sig – significant, OS – overall survival, PFS – progression free survival
